# The complete plastid genome sequence of *Rheum wittrockii* (Polygonaceae), endangered species of Kazakhstan

**DOI:** 10.1080/23802359.2017.1361359

**Published:** 2017-08-08

**Authors:** Shynar S. Dagarova, Gulnara T. Sitpayeva, Jae-Hong Pak, Jung Sung Kim

**Affiliations:** a Faculty of Biology and Biotechnology, al-Farabi Kazakh National University, Almaty, Kazakhstan;; b RSE “Institute of Botany and Phytointroduction” CS MES of Republic, Almaty, Kazakhstan;; c Research Institute for Dokdo and Ulleungdo Island, Kyungpook National University, Daegu, Republic of Korea;; d Department of Biology, Kyungpook National University, Daegu, Republic of Korea

**Keywords:** *Rheum wittrockii*, chloroplast genome, *rpl32*, *rpoA*

## Abstract

The complete chloroplast (cp) genome sequence of *Rheum wittrockii,* an endangered and medicinal plant of Kazakhstan, was firstly determined in the present study. It was 159,051 bp and contained a large single copy region (84,750 bp) and a small single copy region (12,999 bp) which were separated by two inverted repeat regions (30,651 bp). In total, 131 genes were identified and they were consisted of 79 coding genes, 8 rRNA genes, and 36 tRNA genes. *rpl23* was pseudogenes due to sequence substitution. Among 23 genes containing introns, *rps12* and *ycf3* contained two introns and the rest had just one intron. Comparing to Chinese *R. palmatum* chloroplast genome*, rpl32* and *rpoA* gene were shortened due to 1bp and 7bp deletion at poly-T and poly-A, respectively.

*Rheum* L. (Polygonaceae) is mainly found in Asia including Kazakhstan (Baytenov 2001). The recent molecular phylogenetic research revealed that this genus had undergone rapid radiation (Wang et al. 2005; Sun et al. 2012; Wan et al. 2014). In Kazakhstan, *R. wittrockii* Lundstr is one of the endangered plants and is distinguished by large root leave with long petiole (Kokoreva et al. 2013). Traditionally it was used as a Chinese medicine (Barney and Hummer 2012). Even its biological importance and economic usage in Kazakhstan, there is just a little understand of this plant species.

We collected the plant material from the gorge Turgen, Ili Alatau mountains, Enbekshikazakh, Kazakhstan and the voucher was deposited in the herbarium of al-Farabi Kazakh National University. Complete chloroplast genome of *R. wittrockii* (KY985269) was sequenced by HiSeq4000 of Illumina. Totally 44,454,524 paired-end reads (2 × 151bp) were obtained and 3,626,189 reads were assembled to the reference chloroplast genome of *R. palmataum* (Fan et al. 2016) after reads end trimming with an error probability limit of .01. And then assembled reads were *de novo* assembled using the Geneious assembler. Using the assembled contigs, we conducted to align and repeat the procedure up to make a single contig. Complete chloroplast genome was annotated using Geneious 10.1.2 (Kearse et al. 2012) with manual correction and tRNAScan-SE (Lowe and Eddy 1997) for tRNA gene.

It was typical circular form with 159,051 bp in length and comprised a large single copy region (LSC, 84,750 bp), a small single copy region (SSC, 12,999 bp), and two inverted repeat regions (IR, 30,651 bp). It was composed of 131 genes and they were identified 79 coding genes, 8 rRNA genes, 36 tRNA genes, 1 pseudogenes. *rpl23* was pseudogenes due to substitution in third and first base of start and stop codon position, respectively. Interestingly, we found that *rpl32* and *rpoA* gene were shortened comparing to Chinese *R. palmatum* because of 1bp and 7bp deletion at poly-T and poly-A, respectively even though both species were closer to each other in the tree (Figure 1). The present data will be applicable to the further study for understanding the genetic diversity of genus *Rheum* and phylogenomic study for Polygonaceae.

**Figure 1.**
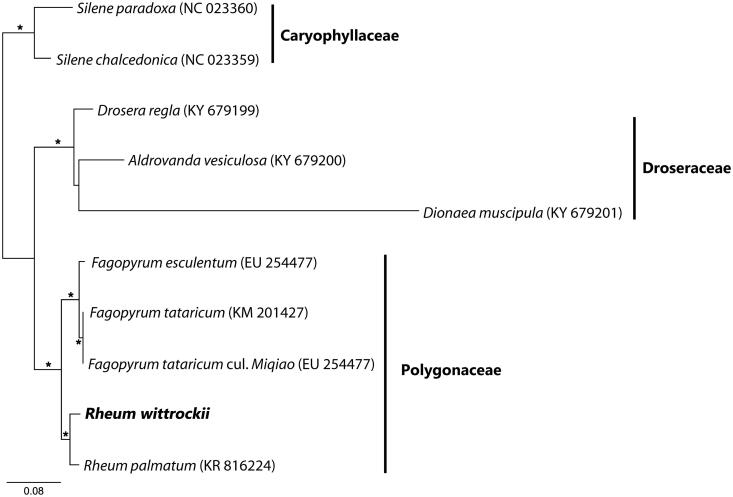
Phylogenetic tree of *Rheum writtrockii* and related taxa using the complete chloroplast genome sequences. Asterisk indicates 100% bootstrap values.
